# Assigning goal-probability value to high intensity runs in football

**DOI:** 10.1371/journal.pone.0308749

**Published:** 2024-09-12

**Authors:** Sam Gregory, Sam Robertson, Robert Aughey, Bartholomew Spencer, Jeremy Alexander

**Affiliations:** 1 Institute for Health & Sport, Victoria University, Melbourne, Australia; 2 Inter Miami CF, Miami, Florida, United States of America; Universidade de Evora, PORTUGAL

## Abstract

High intensity run counts—defined as the number of runs where a player reaches and maintains a speed above a certain threshold—are a popular football running statistic in sport science research. While the high intensity run number gives an insight into the volume or intensity of a player’s work rate it does not give any indication about the effectiveness of their runs or whether or not they provided value to the team. To provide the missing context of value this research borrows the concept of value models from sports analytics which assign continuous values to each frame of optical tracking data. In this research the value model takes the form of goal-probability for the in-possession team. By aligning the value model with high intensity runs this research identifies positive correlations between speed and acceleration with high value runs, as well as a negative correlation between tortuosity (a measure of path curvature) and high value runs. There is also a correlation between the number of players making high intensity runs concurrently and the value generated by the team, suggesting a form of movement coordination. Finally positional differences are explored demonstrating that attacking players make more in-possession high intensity runs when goal probability is high, whereas defensive players make more out-of-possession high intensity runs while goal probability is high. By assigning value to high-intensity runs practitioners are able to add new layers of context to traditional sport science metrics and answer more nuanced questions.

## Introduction

Player movement in football (and most team sports) has predominantly been analysed through two separate dimensions: physical output and player tactical performance. Sport science has often focused on questions relating to the physical element including player load and movement patterns [1], whereas the subdiscipline of sports analytics has explored questions around player and team tactical performance, action value attribution, and overall player value [2, 3]. In recent years in the sports analytics field, particularly in football, there has been an increased focus on value models derived from both event data (data sampled at uneven intervals every time there is an on-ball action typically only encoding the information about the player making the action) [4] and tracking data (data sampled at consistent intervals typically several times per second encoding the location of all players and the ball) [3].

The idea of value in most team invasion sports is generally considered in terms of the probability of scoring or conceding [3]. In sports like basketball, rugby, American football and Australian football (AF) where there are different point totals the value function being optimised is a continuous scoring function of expected points rather than a binary goal scoring function [5–7]. Value models were first introduced in the literature by analysing the goal probabilities of a small subset of events, for example shots in the popular expected goal model which assigns each shot a goal probability based on its features such as distance to goal and body part [8]. This expected goal framework has also been used in other sports such as ice hockey [9] and extended to include multiple different scoring methods in sports such as AF [10].

These early approaches started by considering only the single event before a scoring opportunity but led to the natural extension of moving backwards through event logs from the scoring event to all preceding events in the possession. A common propagation method is using Markov value models, which rely on the assumption that future states depend only on information encoded in the current state and not any past states [11]. Treating team ball (or puck) possessions as Markov chains has been used to create value models across multiple sports [12–15]. Other approaches have considered events individually instead of as part of a chain. Examples include using *k*-nearest neighbours to identify events with similar characteristics and evaluate the outcomes [16] or tree-based machine learning models to estimate values on user-defined feature sets [17].

In more recent literature value models have been extended to incorporate tracking data sets. Notably, Cervone et al 2014 [5] created a metric called Expected Possession Value (EPV) which takes into account the continuous (or near continuous frequently sampled) locations of all players on the court to calculate the point expectation of a given possession in basketball at the same sampling frequency as the tracking data. A similar approach was applied using football tracking data in Fernandez et al 2021 [18], a model which employed a series of convolutional neural networks alongside several other modelling techniques. A key input to the model in Fernandez et al 2021 [18] was the output of a pitch control model. Pitch control models are tracking data analyses that use spatial control as the target variable instead of goal probability [19–21]. Similar to the approach of Decroos et al 2019 [17], but extended to tracking data, Spencer et al 2019 [6] calculated a feature set on each individual frame of AF tracking data and made expected point predictions on each frame.

As value models have moved away from an event-data based architecture (typically a new value propagated every couple of seconds) to a tracking-data based model they have the added advantage of not only encompassing greater information and data about the state of play, but also are continuous at the frame rate of the tracking data (anywhere from 5–30 Hz). Physical metrics used in sport science research are typically extracted from tracking-data, which means tracking-data based value models allow for a direct comparison between physical metrics and value models. The broad availability of tracking data and associated research has led to a new field of literature bringing together sport science and sports analytics research through tracking data analyses.

Modern sport science literature has questioned the value of physical metrics without context [22, 23] and used analytics tools to help put these physical performance metrics in context [24, 25]. Many studies have used playing position as a proxy for tactical context and analysed the different physical metric outputs by position [26–28], as well as overall team outputs by playing formation [29]. Bradley and Noakes 2013 [28] identified significant scoreline effects on high intensity running. Gregory et al 2022 [24] extended these analyses of physical metrics from playing position and scoreline to more time-varying dimensions of phases of play and win probabilities. Bradley and Ade 2018 [22] introduced the idea of analysing player physical outputs across manually tagged run types or actions. Llana et al 2022 [25] used the value model from Fernandez et al 2021 [18] to answer a series of potential questions that incorporate both physical and tactical elements.

In the same vein of using tools from analytics to provide context to existing sports science metrics, this study introduces a new approach assigning value to player high speed runs from a team-value perspective. It does so by considering value alongside the physical movement profiles of specific runs. A machine learning based (*xgboost*) value model is used to calculate continuous goal scoring probabilities for the in-possession team. The change in value accrued over the course of a high speed run is then matched with various movement profile characteristics of the run. In addition to looking at the relationship between movement metrics and value on a run-by-run basis, more macro-level topics are explored such as the player positional differences in high speed run values, the potential effects of team coordinated movement and other applications.

## Methodology

Research procedures reported in this study were approved by the Victoria University Human Research Ethics Committee (application number: HRE21-062).

### Data

The data used in this paper are full event and tracking data from the 2022 Major League Soccer (MLS) Regular Season (a total of 475 matches). The event data is collected by StatsPerform and the tracking data by Second Spectrum (tracking data). The Second Spectrum tracking data has received FIFA’s EPTS certification for 2022–2024 and is rated well-above industry standard for velocity and positional tracking accuracy in football [30]. The tracking data provided were sampled at a rate of 25 frames per second and include all player and ball locations. However, for the purpose of this analysis the tracking data were downsampled to a rate of 5 Hz for computational efficiency by selecting every fifth frame. This procedure (and others) of downsampling maintains data fidelity for a variety of analysis purposes [31–33]. The data were aligned by the vendor so that each event corresponds to a particular frame in the tracking data.

### Value model

The events were split into a series of possessions defined by the provider as:

*A sequence of time during which control of the ball doesn’t change. A single team’s possession can continue through the defending team’s clearance or deflection*.

*Possessions are always split on dead balls (dead balls always interrupt possessions*, *regardless of who retains or gains possession as a result of the dead ball)* [34].

For the purposes of assigning value, a possession (*p*) can end with one of two outcomes: the team in-possession scores (G_p_ = 1) or the team in-possession does not score (G_p_ = 0).


$${\text{G}}_{\text{p}}\in \left\{0,1\right\}$$ (1)

If possession *p* has F frames the outcome variable G_p_ can be estimated as the current value on each frame *f* as V_f_(p) where V_f_(p) is the value of the possession *p* at frame *f*.


$$f\in \left[0,\text{F}\right]$$ (2)

This value is estimated using the feature set calculated on every frame *f* referred to as **X**_f_. The function used to estimate the value on each individual frame is an xgboost (Extreme Gradient Boosting) binary classifier [35], a scalable boosted tree learning method. This estimator is referred to with the function Θ. Hyperparameters were tuned using a random search algorithm across a range of possible values.


$${\text{V}}_{\text{f}}\left(\text{p}\right)=\Theta \left(X_{\text{f}}\right)$$ (3)

The value model was fit on a random subsample of 150 matches at a downsampled rate of 5 frames per second for reasons of computation efficiency.

The following features were used to create the feature matrix **X**_f_ on each individual frame. These features and the overall value function were calculated on a downsampled version of the tracking data of 5 frames per second. The feature set closely reflects the features chosen in Spencer et al 2019 [6] adapted from AF to football. Note that any distance related features take into account only x,y coordinates on a 2D plane with no player or ball height considerations.

#### Overall features

These features are unique to each frame and not calculated separately for the in-possession and out-of-possession team.

*Euclidean distance to the goal*. The Euclidean distance in metres from the ball to the centre of the out-of-possession team’s goal.

*Angle to the goal*. The angle (in radians) from the ball to the out-of-possession team’s goal relative to the touchline (ex. if the ball is in the centre of the field the angle from the touchline to the goal is 90 degrees or ~1.57 radians, if it is at the corner flag the angle would be 180 degrees and ~3.14 radians).

#### In and out of possession features

These features are calculated for both the in and out of possession teams and are separate features for each team in the model.

*Team centroid X and Y coordinates*. The centroid of all of the x, y coordinates (in metres) of the non-goalkeeper players on each team. The x, y coordinates are two separate features.

*Euclidian distance from ball to team centroid*. The distance in metres from each team’s centroid to the ball.

Ball x-coordinate *Distance from Team Centroid*: The distance in metres from the along the x-axis (goal line to goal line) of the ball to each team’s centroid.

Number *of Players Between Ball Line and Out-of-Possession Team’s Goal*: A count of the number of players on each team between the ball and the out-of-possession team’s goal.

*Team area*. The area in metres squared of the convex hull made by the x, y locations of the non-goalkeeper players on each team.

*Team displacement (1 second)*. The average displacement—Euclidian distance between the start and end locations—of all the players on each team in the previous second.

*Team displacement (5 seconds)*. The average displacement of all the players on each team in the previous five seconds.

*Team angular displacement (1 second)*. The average angular displacement between consecutive movement vectors of each player. i.e., The vector formed by the coordinates of the player’s location two seconds ago and one second ago relative to the vector formed by the coordinates of the player’s location one second ago and current location.

*Team pitch control*. The amount of surface area “controlled” by each team taking into account player locations and movement vectors [19]. In this case this feature is analogous to the total density of each team.

**Fig 1:**
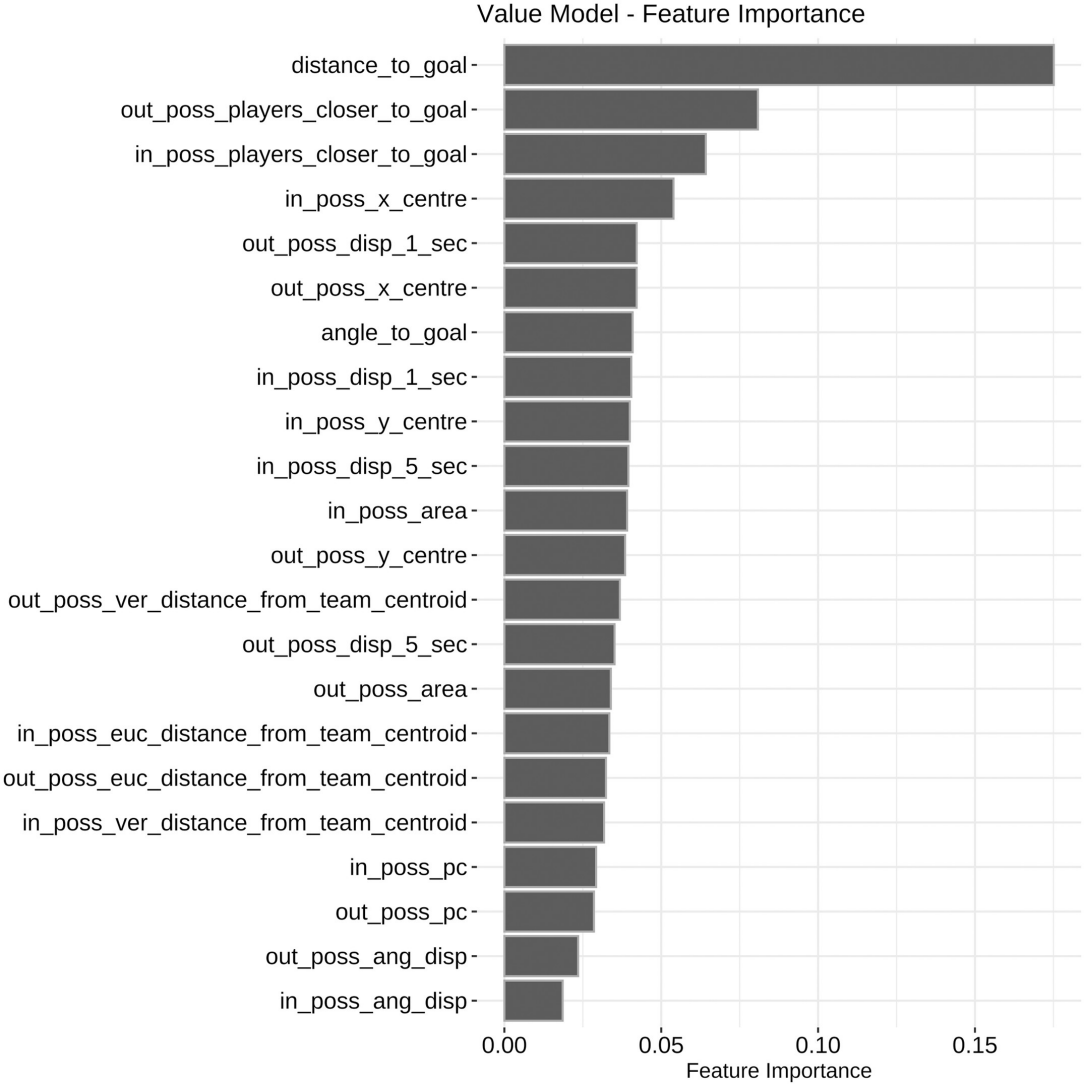
Feature importance of all features in xgboost value model. Distance of the ball to goal is the highest importance feature followed by out-of-possession players then in-possession players closer to the defending team’s goal than the ball.

Fig 1 shows the relative feature importance in the final model fit.

Distance from the ball to the goal is by far the most important feature followed by out-of-possession players closer to the defending goal than the ball and then in-possession players closer. There are several interesting trends comparing the feature importance of in and out-of-possession features; for example, the “x-coordinate” centre is more important for the in-possession team than the out-of-possession team suggesting how high up the pitch the attacking players are has a larger effect on goal scoring probability. Conversely, the displacement of the out-of-possession team has a higher importance than that of the in-possession team suggesting movement by the out-of-possession team is a better indicator of goalscoring probability (potentially as a proxy for defensive disorder).

The predictions were made on every frame at the downsampled tracking data rate of 5 Hz using the features and framework outlined above. The independent nature of the predictions (each goal probability is calculated based on the information of a single frame only) led to momentary blips or jumps in the value predictions which are unlikely to be reflective of true changes in goalscoring probability. To mitigate these blips a 3-frame triangular moving average was applied to the predictions so that each prediction was the mean of the frame itself and the frames directly preceding and succeeding.

### Run value

Runs were defined as any period of sustained running by a player at a speed of over 5.5 m·s^-1^ for at least one second, terminating once the player drops below this speed [36]. The value accrued over the course of a run was calculated as the total change in the in-possession team’s value over that run.

Consider a run *r* which lasts for n frames beginning at frame *f*_*1*_ and ending on frame *f*_*n*._ The value on each frame *f*_*i*_ as calculated by the value model is represented by *V(f*_*i*_*)*. The value of the run *V(r)* is calculated as the average value of all the frame values *V(f*_*i*_*) minus* the value at the start of the run.


$$\text{V}\left(\text{r}\right)=\left(1/\text{n}\right){\sum^{\text{n}}}_{\text{i}=1}\text{V}\left({\text{f}}_{\text{i}}\right)-\text{V}\left({\text{f}}_{1}\right)$$ (4)

This approach is different than how value is assigned in several other models to on-ball events which just take the difference in value at the start and end of the action (ie. V(f_n_)—V(f_1_)) [12, 14]. The rationale for this new approach is twofold: firstly, runs are continuous actions that occur over several seconds unlike instantaneous events such as shots and passes, and secondly the value itself may impact the decision to end the run.

Consider a player who starts to accelerate to initiate or join a counter attack, that counter attack may lead to a high probability of scoring thus the run itself was of high value but once the counter attacking opportunity ends the player will de-accelerate and end their run. Using the approach V(f_n_)—V(f_1_) the run may have a negligible or even negative value even though the team had a high chance of scoring during (and potentially even because of) the run. By considering the average value accrued over the course of the run the high value of that run is better captured using the calculation in formula (4).

**Fig 2:**
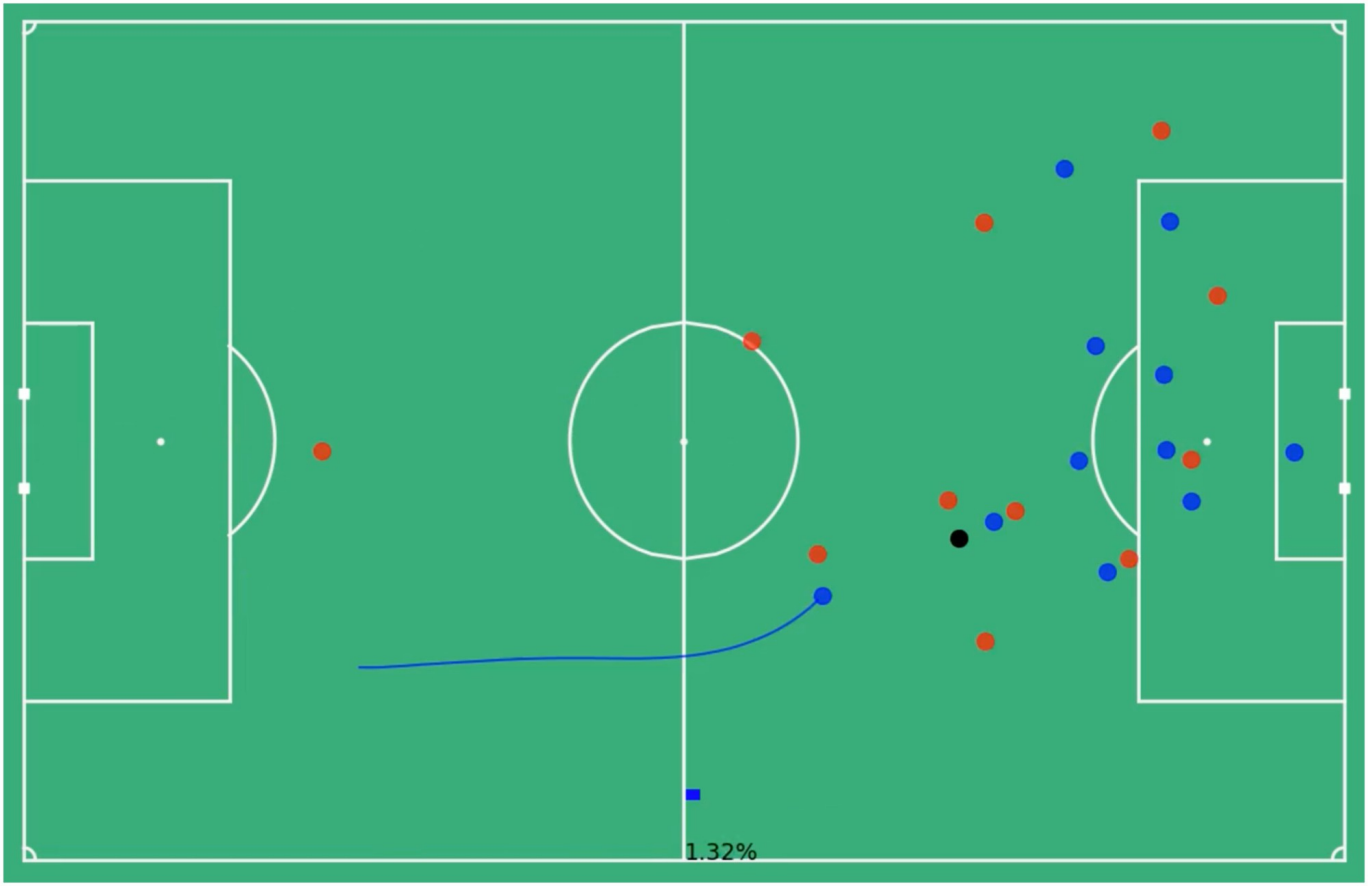
Start of run player positions, 1.32% goal probability. The still image of the start of a run, the blue dots represent the locations of the in-possession players, the red dots the out-of-possession players and the black dot the ball. The value model predicts the blue team has a 1.32% chance of scoring at this moment the blue player is about to begin his run along the highlighted trajectory.

**Fig 3:**
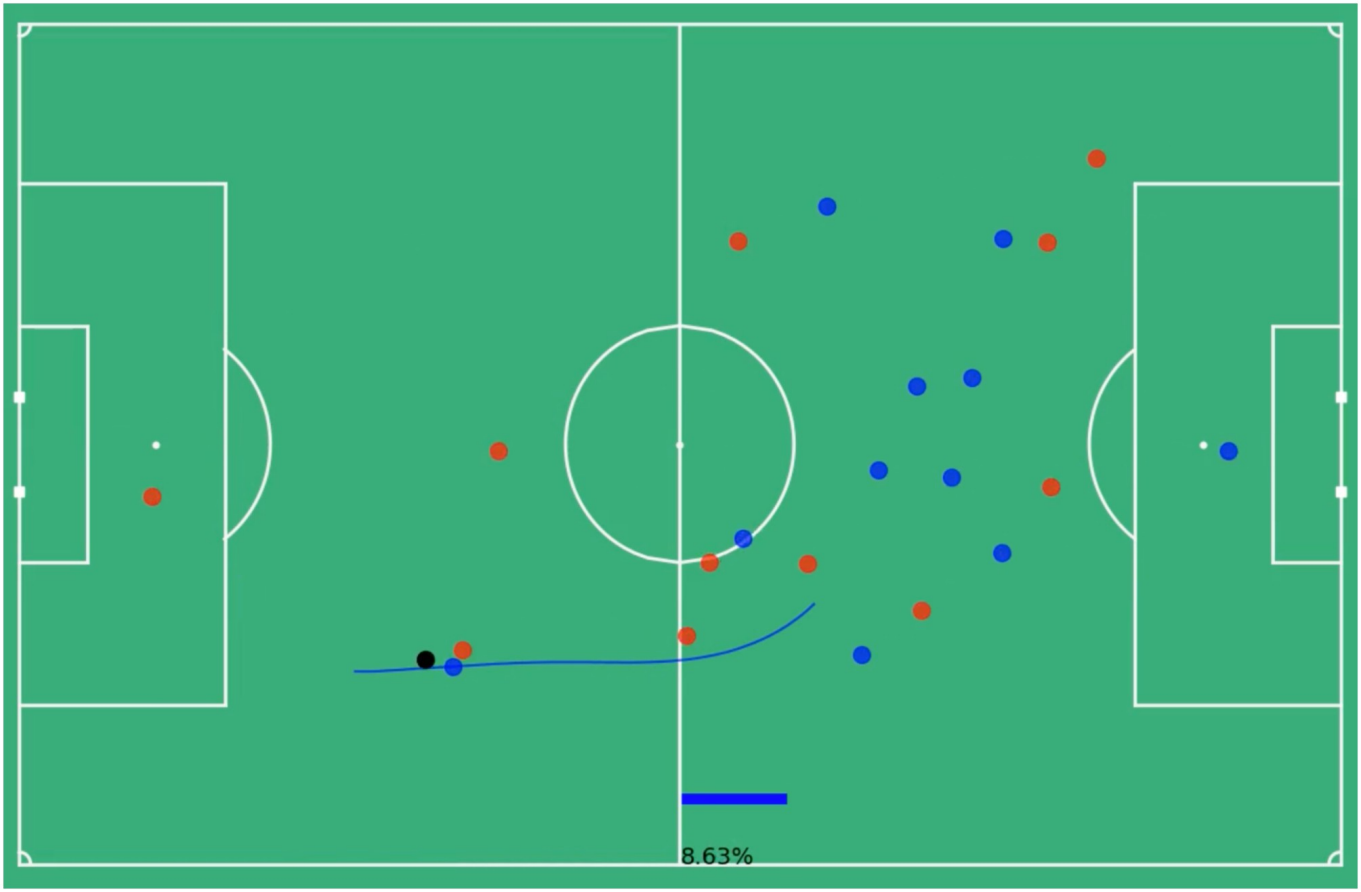
Highest value moment in run player positions, 8.63% goal probability. The still image of near the end of a run, the blue dots represent the locations of the in-possession players, the red dots the out-of-possession players and the black dot the ball. The value model predicts the blue team has an 8.63% chance of scoring at this moment the blue player is nearing the end of his run along the highlighted trajectory. This is the highest probability of scoring the blue team has over the course of this run.

**Fig 4:**
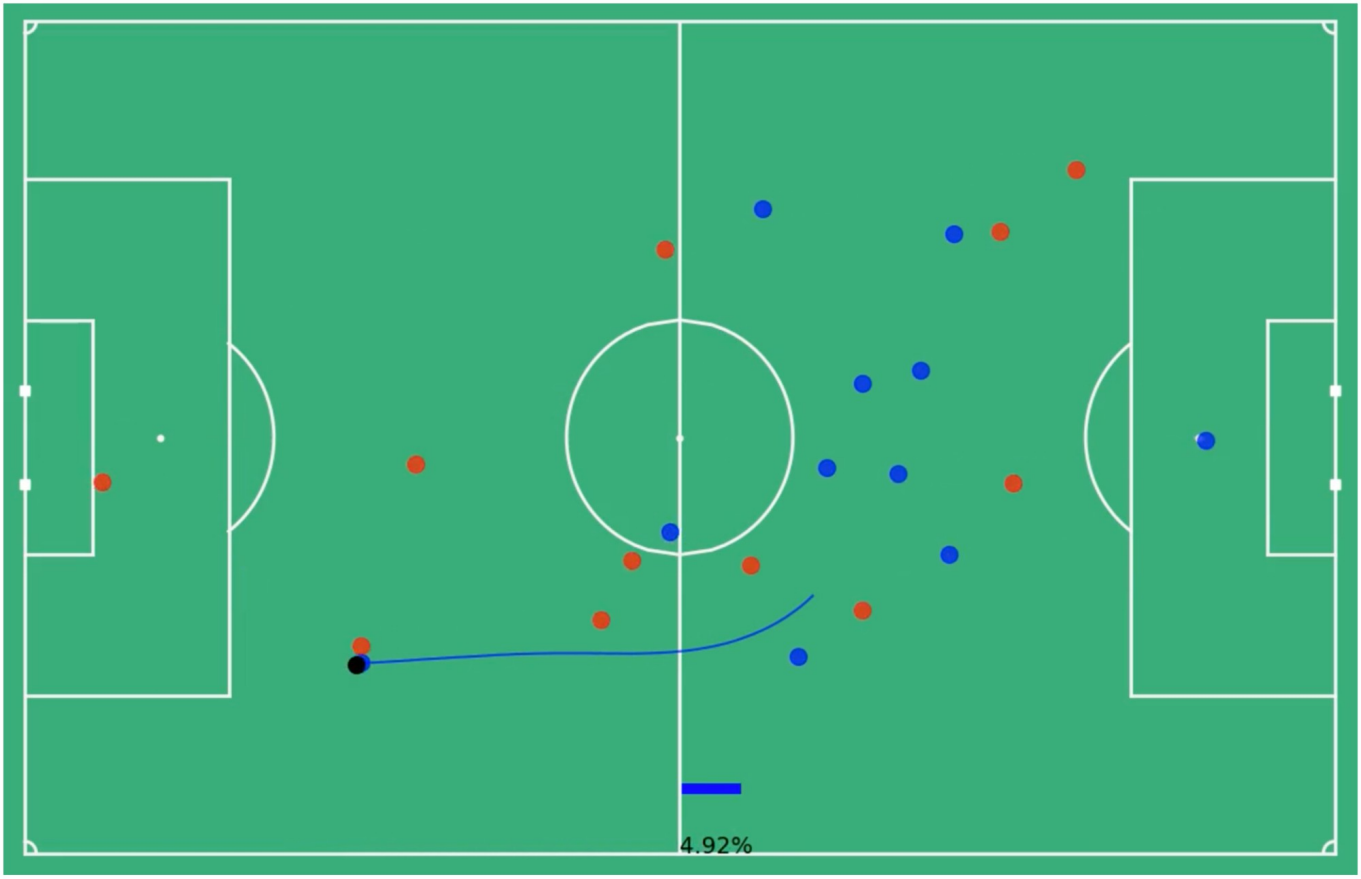
End of run player positions, 4.92% goal probability. The still image of the end of a run, the blue dots represent the locations of the in-possession players, the red dots the out-of-possession players and the black dot the ball. The value model predicts the blue team has a 4.92% chance of scoring at this moment the blue player is at the end of his run along the highlighted trajectory.

Consider the run outlined in the Figs below. This run lasts for 4.88 seconds or 122 frames, which is then downsampled to 24 frames. Beginning in Fig 2 the in-possession blue team has a goal probability of 0.0132 or a 1.32% chance of scoring. The blue dots represent in-possession players, the red dots out-of-possession players and the black dot the ball. The future trajectory of the run is outlined in blue and the player at the tail of that trajectory is the one making the run. Near the end of his run in Fig 3 the blue team reaches its highest probability of scoring of 8.63%. By the time the player ends his run in Fig 4 that probability has dropped to 4.92%. The average goal probability of the run over the 24 downsampled frames is 4.62%, so using the formula above (4) this particular run has a total value accrued of 0.033 (3.3% goal probability).

For a further demonstration of both the run value calculations and the value model itself refer to the following video which highlights the 10 highest and lowest value accrued runs both in and out-of-possession from a sample game [link redacted for blind reviewing purposes].

### Movement metrics

This paper employed three different movement metrics which are calculated on a frame-by-frame level, however they are reported as run-level values. As outlined in above all high speed runs are at least one second so contain at minimum five frames from the downsampled tracking data. The reported value for each run is the maximum value reached over the course of the run. This methodology is consistent for the three movement metrics described below.

#### Speed (m·s^-1^)

Speed calculations are provided by the vendor and are based on a regression line through the five frames centred around the current frame (2 before and 2 after), reported in (m·s^-1^) [36]. These five frame windows are calculated directly from the vendor so use the full 25 Hz data not the downsampled 5 Hz data.

#### Acceleration (m·s^-2^)

Accelerations are the first derivative of speed calculations with no additional smoothing, beyond those used in the speed calculations, and are reported in metres per second squared (m·s^-2^). Note that because the vendor provided speed calculations are calculated at a 25 Hz rate the acceleration calculations implicitly make use of the full data set, but are only directly calculated at the downsampled 5 Hz rate.

#### Tortuosity

In addition to more traditional measures of speed and acceleration this research also uses the path analysis metric of tortuosity, which has been used in other fields such as ecology to measure the curvature object trajectories [37] and has recently emerging as a more popular metric for player path analysis in sport [24, 38].

**Fig 5:**
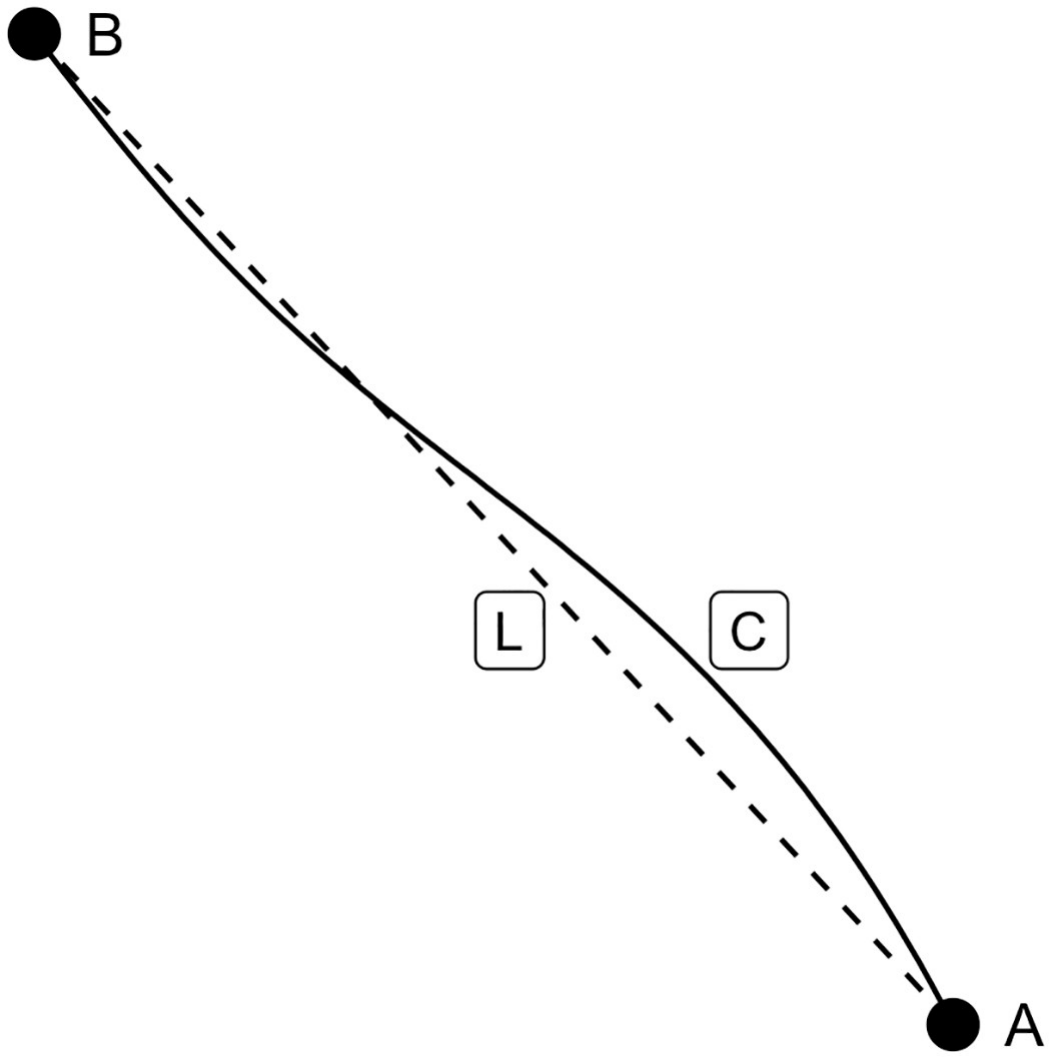
Tortuosity example, player moving along path C. In this example the player moves along the path C from point A to point B over three seconds. Tortuosity is a measure of the ratio of the length of the actual path travelled C and the length of hypothetical straight line distance L.

The tortuosity measure used here is a simple measure of how curved a player’s high speed run is. The metric is calculated as the ratio of real distance travelled to straight-line distance travelled bounded on the scale *[0,1]*. Tortuosity takes into account the path taken by the player in the previous three second window and calculated on each frame. An example calculation is shown in Fig 5.

In Fig 5 the player moves along line C from point A to B, where L is the quickest possible path from A to B. Tortuosity is calculated as follows:

$$Tortuosity=\;1\;-\frac{Length\left(L\right)}{Length\left(C\right)}$$ (5)

The closer the tortuosity measure is to 0 the more similar the lines L and C are and the less arched the actual run C is.

### Run-value analysis

The value accrued over high speed runs are first summarised and then compared over several dimensions and cross-variants.

1. The differences in value accrued based on the physical metric attributes of the high speed runs (speed, acceleration, tortuosity).
2. The difference in average value accrued in high speed runs by playing position.
3. The effect of coordination on value accrued in high speed runs, ie. is there an effect or correlation between multiple players on the same team making high speed runs concurrently.

### Player case study and sample outputs

A player-specific case study is presented to demonstrate how analysing the value of a player’s runs throughout a match alongside the physical attributes of those runs can add an additional layer of insight and be used in an applied context. A sample table is also included analogous to the tables that are typically included in physical post-match reports by sport scientists, demonstrating how value accrual could be used to make them more impactful.

## Results and analysis

### Run value distributions

Over the course of the 475 matches there were a total of 628,186 high-speed runs for an average of 1322.5 per match. Which translates to approximately 60.1 per player per match for starters who play the full 90 minutes.

Runs can either be in-possession only, out-of-possession only, overlap between the two (run starts with the team in-possession and ends with the team out-of-possession or vice-versa), or involve neither team in possession. Approximately 6.0% of runs fall into overlap of in and out-of-possession runs, because this analysis treats the value generated from in and out of possession runs separately these runs are split in the analysis and each part is analysed separately. As a result, the sum of all in-possession runs and all out-of-possession runs is greater than the total sum of all runs. 3.8% of the runs occur when neither team is in-possession and these are ignored in the value analysis.

Of the total number of runs 56.5% include at least one out-of-possession segment and 45.7% include an out-of-possession segment. The total run counts are summarised in Table 1. Table 2 highlights the percent of runs by time duration.

**Table 1:** Summary of all runs in sample split by in-possession and out-of-possession, note that some runs include both in and out of possession segments and some include neither in nor out of possession segments.

	Total Runs	Per Match	~Per Player Match
**In-Possession**	286875	603.95	27.45
**Out-of-Possession**	355224	747.84	33.99
**Overall**	628186	1322.5	60.11

**Table 2:** Percent of all runs in the sample that fit into each time duration bucket.

*1–2 Seconds*	*2–3 Seconds*	*3–4 Seconds*	*4–5 Seconds*	*5–6 Seconds*	*6–7 Seconds*	*7+ Seconds*
*53*.*3%*	*26*.*6%*	*11*.*3%*	*5*.*0%*	*2*.*2%*	*0*.*9%*	*0*.*7%*

**Fig 6:**
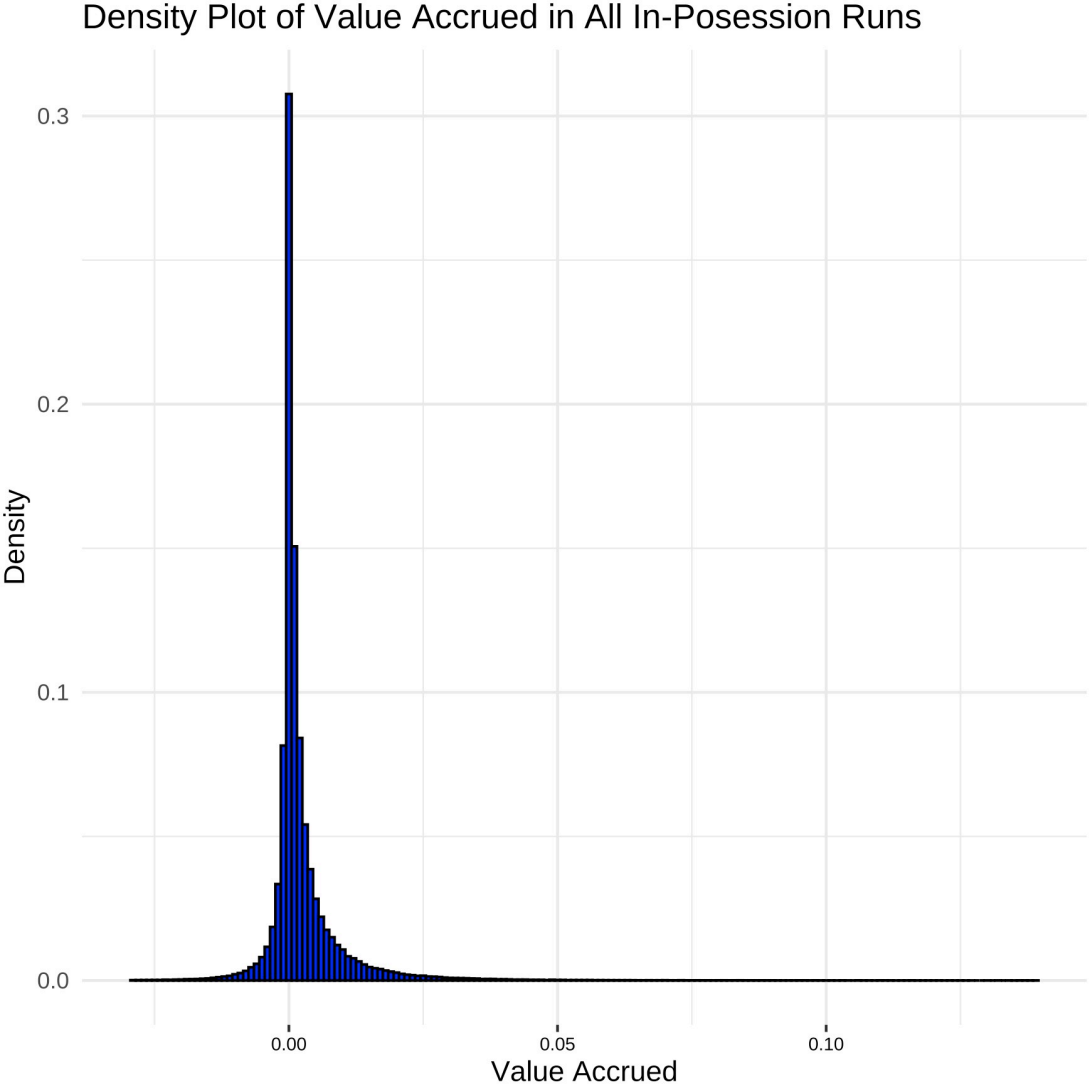
Distribution of value accrued across in-possession runs. Distribution centred at 0 with long tails, cut off at 5th percentile (-0.05 goal probability) and 95th percentile (+0.14 goal probability).

Fig 6 outlines the distribution of value accrued by the in-possession team across all in-possession runs. The distribution is centred at 0, in 79.87% of in-possession high speed runs the team in possession accrues between +/-0.005 value/goal probability.

**Fig 7:**
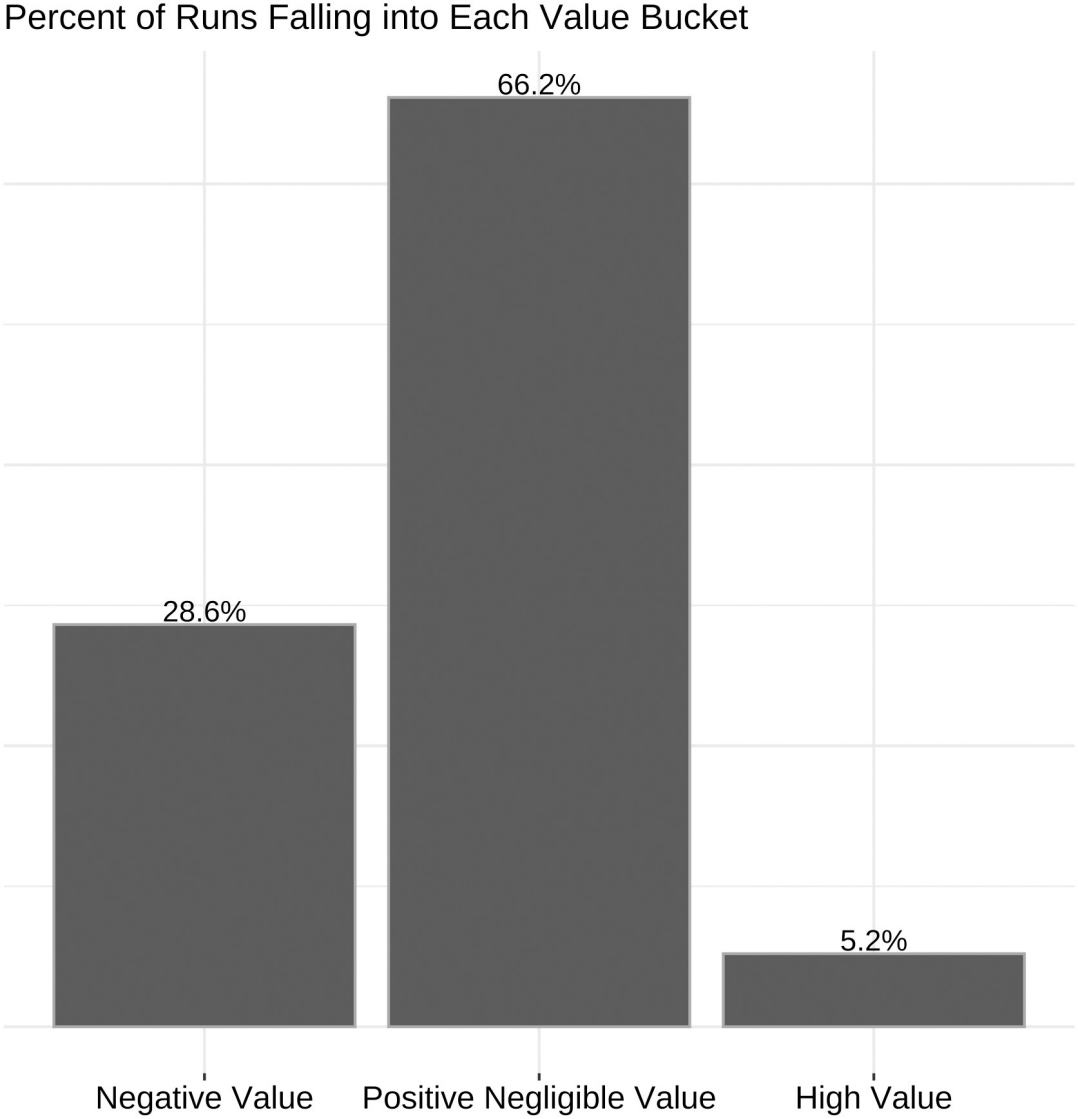
Distribution of value accrued across in-possession runs by bucket. Value buckets are divided into negative value (V<0), positive negligible value (0 ≤ V < 0.014) and high value (V ≥ 0.014).

Because so few of the in-possession runs coincide with major shifts in the probability of the in-possession a team scoring in the rest of the analysis runs are divided into three categories (outlined in Fig 7):

1. Negative Value Runs: any runs where the team accrues negative value (V < 0)
2. High Value Runs: any runs where the in-possession team accrues an average value (using the methodology in 2.3) greater than 0.014 over the course of the run (V ≥ 0.014). 0.014 is chosen as the cut-off because it is the 95th percentile of the value accrued in all in-possession runs.
3. Positive Negligible Value Runs: any runs where the team accrues positive value, but less than the cut off of 0.014 (0 ≤ V < 0.014)

**Fig 8:**
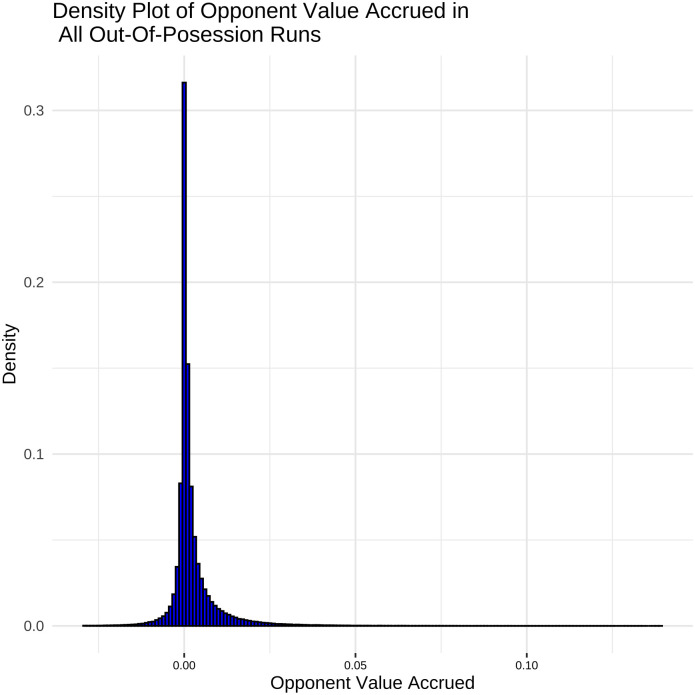
Distribution of opponent value accrued across out-of-possession runs. Distribution centred at 0 with long tails, cut off at 5th percentile (-0.05 opponent goal probability) and 95th percentile (+0.14 opponent goal probability).

The distribution of opposition value accrued over the course of out-of-possession runs in Fig 8 looks very similar to the distribution of own-team value accrued for in-possession runs in Fig 6. As a result the same cut-offs and definitions outlined above are used for out-of-possession runs except using opponent value accrued as the measure instead of own-team value accrued.

### Run-specific differences in value across movement profiles

There are broad, but weak correlations between value accrued and the three movement profile metrics: speed, acceleration and tortuosity. These correlations (r values) are outlined in Table 3.

**Table 3:** Correlation table between player movement metrics and value accrued for in-possession runs and opponent value accrued for out-of-possession runs. Speed is the most correlated with value for both in and out-of-possession runs, then tortuosity (negatively) and then acceleration. The direction of correlation for all metrics for in and out-of-possession runs is the same but the magnitude is higher for in-possession speed and acceleration, but higher for out-of-possession tortuosity.

	Value For In-Possession Runs	Opponent Value For Out-of-Possession Runs
**Speed (m·s-1)**	0.157	0.147
**Acceleration (m·s-2)**	0.050	0.043
**Tortuosity**	-0.079	-0.109

The correlations for value accrued on in-possession runs all run in the same direction as the correlations for opponent value accrued on out-of-possession runs. They are both positive for speed and acceleration and negative for tortuosity. This means that when a player is making a high speed run, regardless of whether that player is in or out-of-possession, higher speeds and accelerations are associated with slightly higher value for the attacking team. The same is true for straighter runs (lower tortuosity).

The relative magnitudes of the correlations are also notable. The speed and acceleration correlations are stronger for the in-possession team, however the (negative) correlation for tortuosity is stronger for the out-of-possession team.

**Fig 9:**
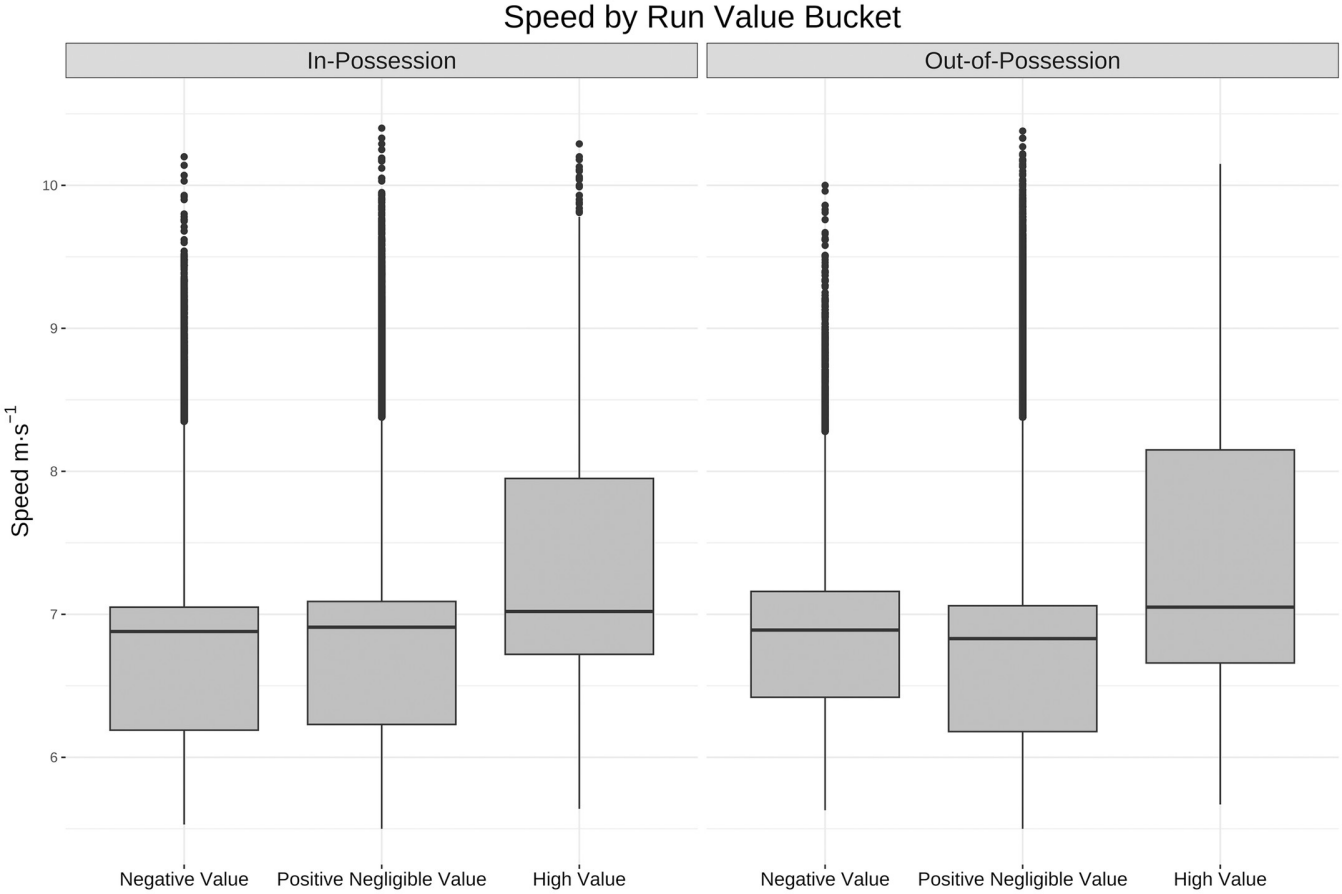
Box and whisker plot for speeds by value bucket. Box and whisker plot (horizontal line showing median, box showing 25th and 75th percentiles, vertical line showing highest and lowest points within 1.5 of inter-quartile range) showing the difference in speed across the three bands of run value. For both in and out-of-possession runs the distribution of max speeds is skewed higher for high value runs.

**Fig 10:**
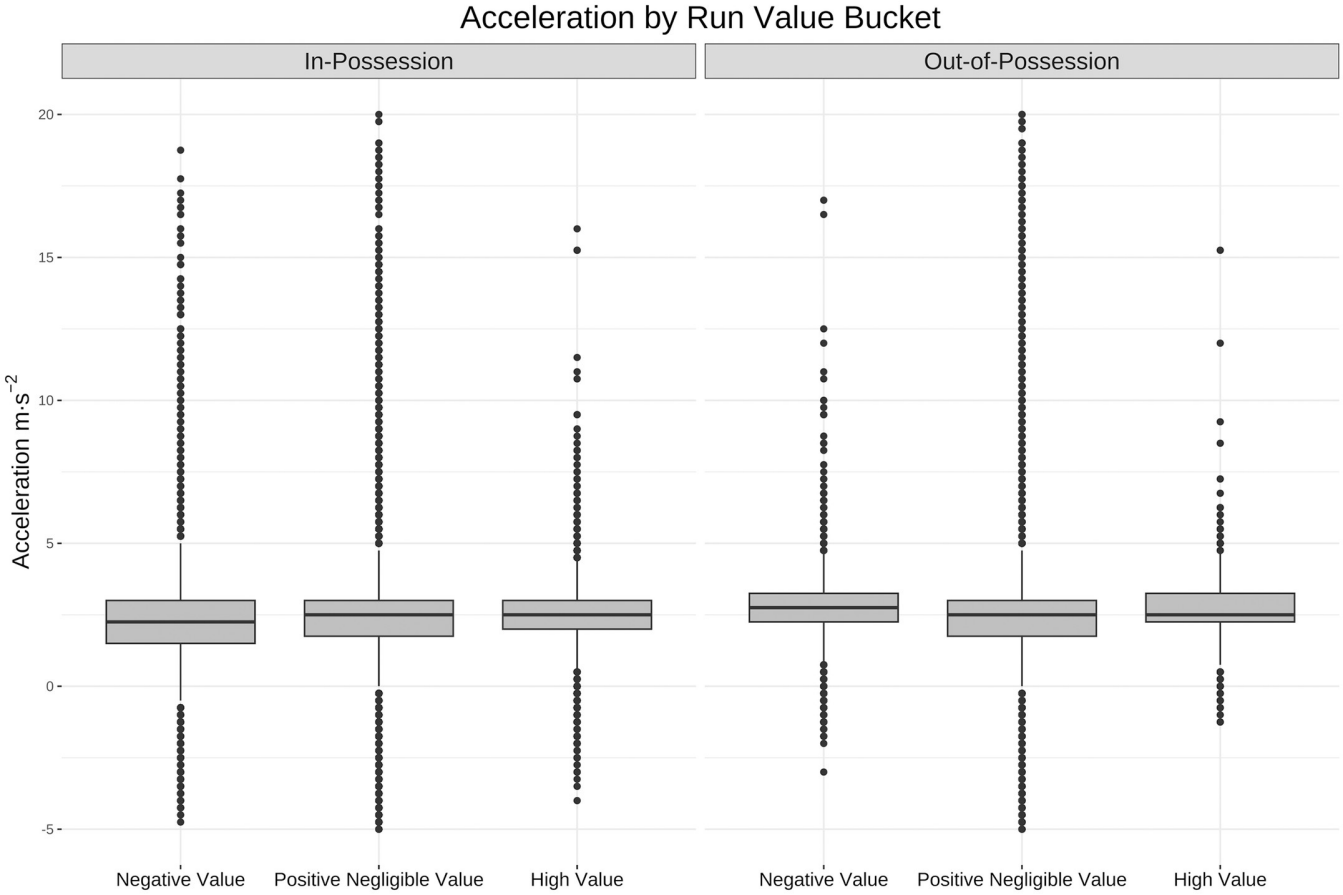
Box and whisker plot for accelerations by value bucket. Box and whisker plot (horizontal line showing median, box showing 25th and 75th percentiles, vertical line showing highest and lowest points within 1.5 of inter-quartile range) showing the difference in acceleration across the three bands of run value. For both in and out-of-possession runs the distribution of max absolute accelerations is skewed slightly higher, but the difference is negligible due to the acceleration outliers (acceleration in this graph is cut off lower than -5 m·s^-2^ and higher than 20 m·s^-2^).

**Fig 11:**
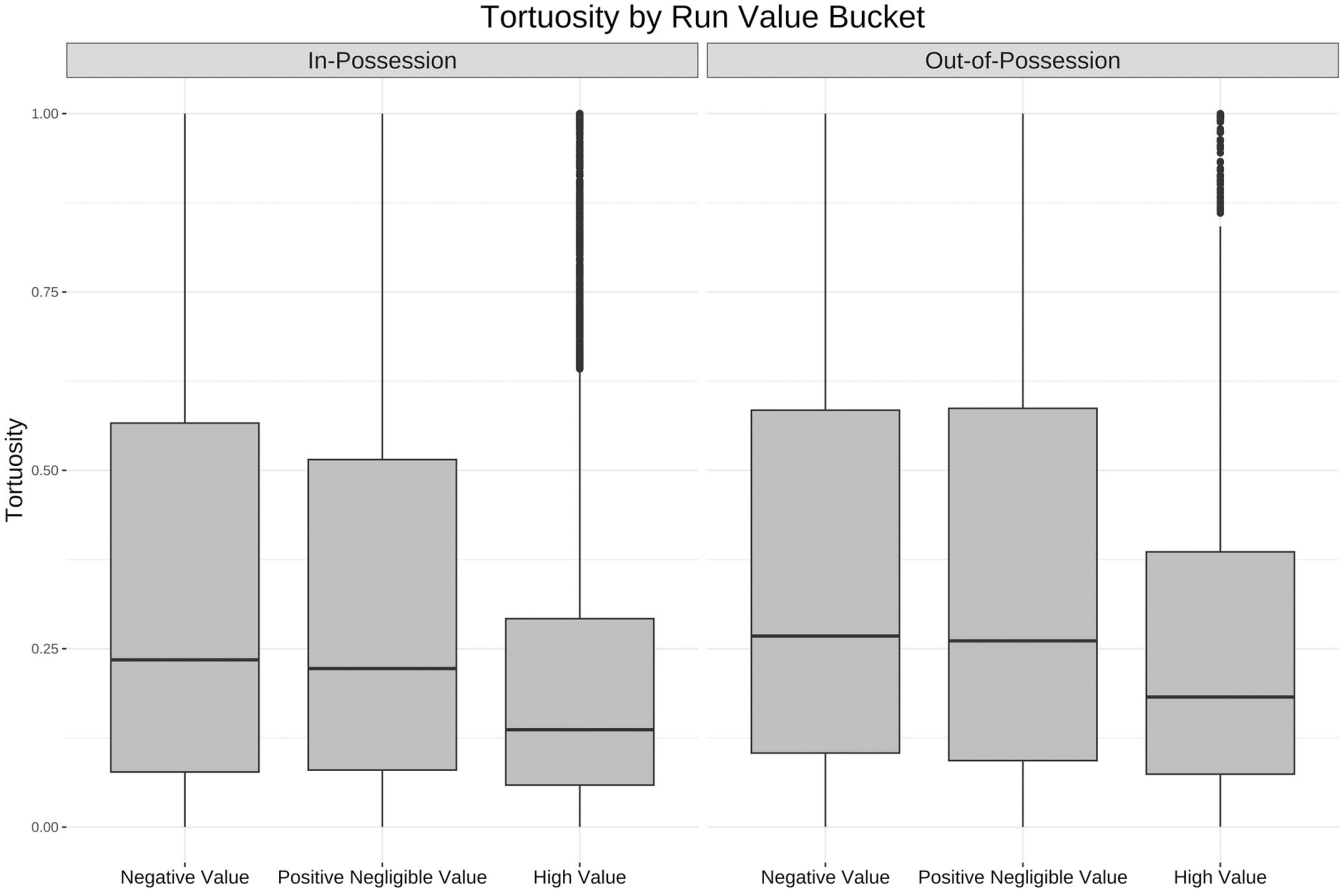
Box and whisker plot for tortuosity by value bucket. Box and whisker plot (horizontal line showing median, box showing 25th and 75th percentiles, vertical line showing highest and lowest points within 1.5 of inter-quartile range) showing the difference in tortuosity across the three bands of run value. For both in and out-of-possession runs the distribution of tortuosity is skewed lower for high value runs.

These correlations are further examined in Figs 9–11 using the buckets established above. The box and whisker plots that follow each have a line at median, a box around the 25th and 75th percentiles and vertical lines or whiskers that extend to the highest and lowest point within 1.5 of the interquartile range (IQR) of the box [39]. These box plots show a much larger difference between movement profiles, particularly in the speed (Fig 9) and tortuosity (Fig 11) distributions, for high value runs than across the other two buckets of negative value and positive negligible value runs.

### Coordination/co-occurrence

**Fig 12:**
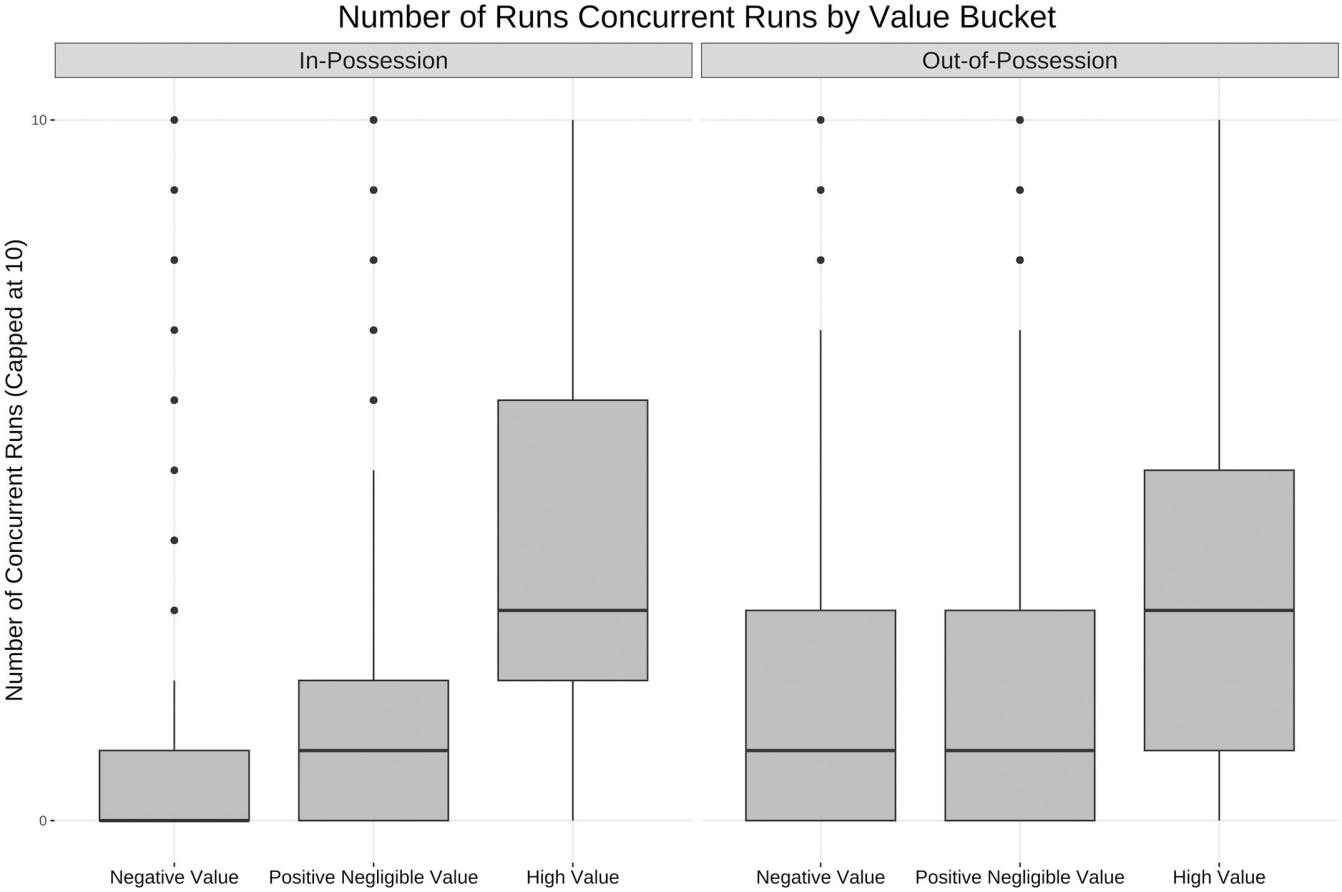
Box and whisker plot for number of concurrent runs by value bucket. Box and whisker plot (horizontal line showing median, box showing 25th and 75th percentiles, vertical line showing highest and lowest points within 1.5 of inter-quartile range) showing the difference in concurrent runs across the three bands of run value. Higher value runs tend to coincide with more players on the same team making high intensity runs, suggesting potential coordination.

The entire team’s value accrued during an individual player run is assigned to each run, this means that while a team is accruing value multiple players can be credited with making high value runs at the same time. Only 35% of runs did not overlap with a single other teammate’s high speed run, on average 2.04 teammates made a concurrent run with each high speed run. Fig 12 shows the distribution of concurrent runs being made by players on the same team. High value runs tend to correspond with more players running both in and out-of-possession.

Note that in Fig 12 the number of concurrent runs is capped at 10, but a run can be concurrent with more than 10 teammate runs despite the fact any one player can only have 10 teammates on the pitch at one time. This is due to the fact a single run by one player can overlap with two or more runs made by the same teammate if that teammate has two separate occurrences of a trajectory meeting the high speed run thresholds overlapping with the time the player in question is making a single high speed run.

### Positional differences in run value

**Fig 13:**
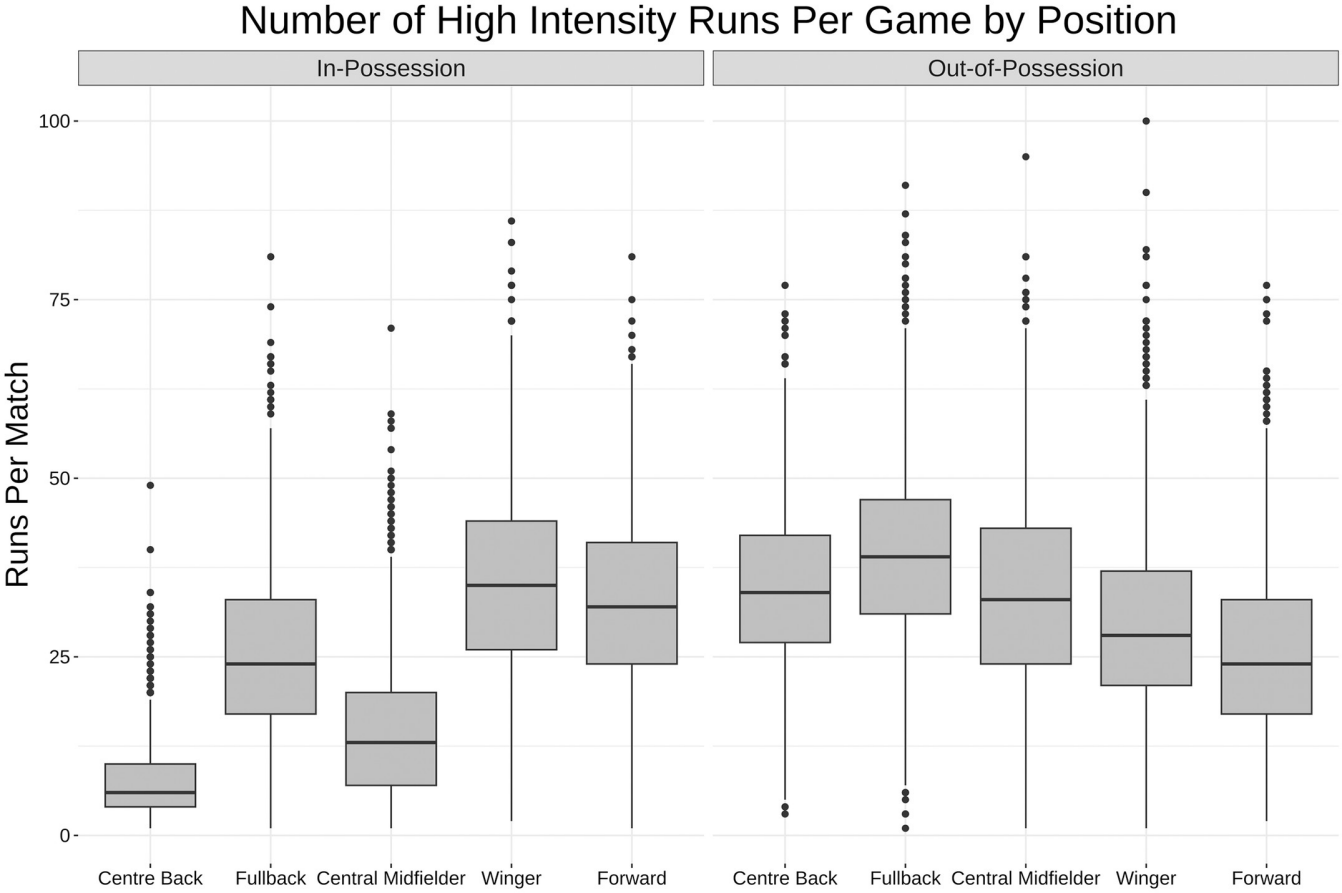
Box and whisker plot for number of high speed runs by position. Box and whisker plot (horizontal line showing median, box showing 25th and 75th percentiles, vertical line showing highest and lowest points within 1.5 of inter-quartile range) showing the difference in number of high speed runs by position. Fullbacks, wingers and forwards make more high intensity runs in-possession, while the distributions out-of-possession are much more evenly distributed across positions.

Fig 13 shows the relative number of high speed runs starting centre backs, fullbacks, central midfielders, wingers and forwards make (for the purposes of this section only any runs made by goalkeepers or substitutes are removed).

Fullbacks, winger and forwards make more in-possession high speed runs, while the distribution of out-of-possession high speed runs is much more evenly distributed, with fullbacks making the most and forwards making the fewest.

**Fig 14:**
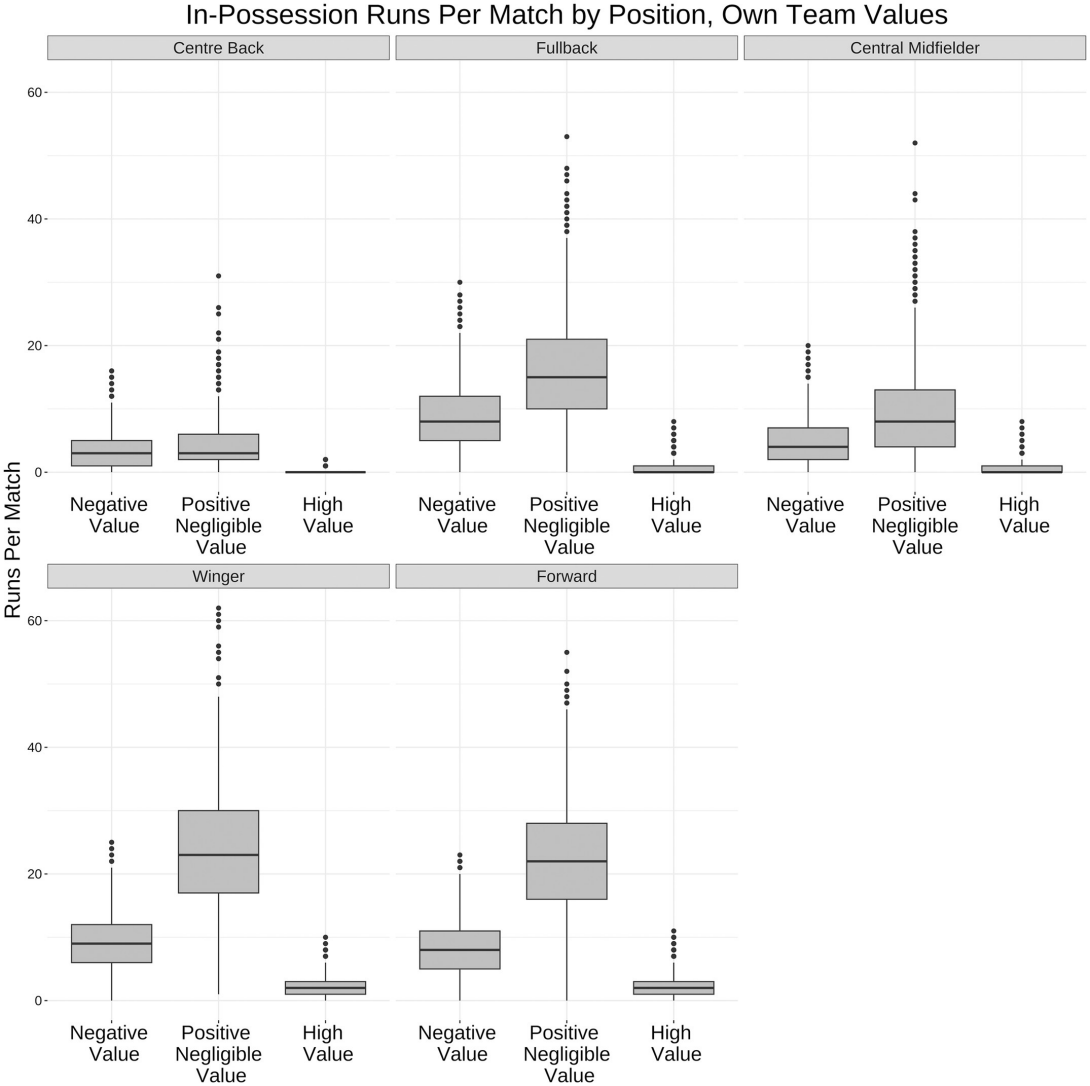
Box and whisker plot for in-possession run value buckets by position. Box and whisker plot (horizontal line showing median, box showing 25th and 75th percentiles, vertical line showing highest and lowest points within 1.5 of inter-quartile range) showing the difference in run value by positions in-possession. Wingers and forwards have the most high value in-possession runs, but also the most negative value runs.

**Fig 15:**
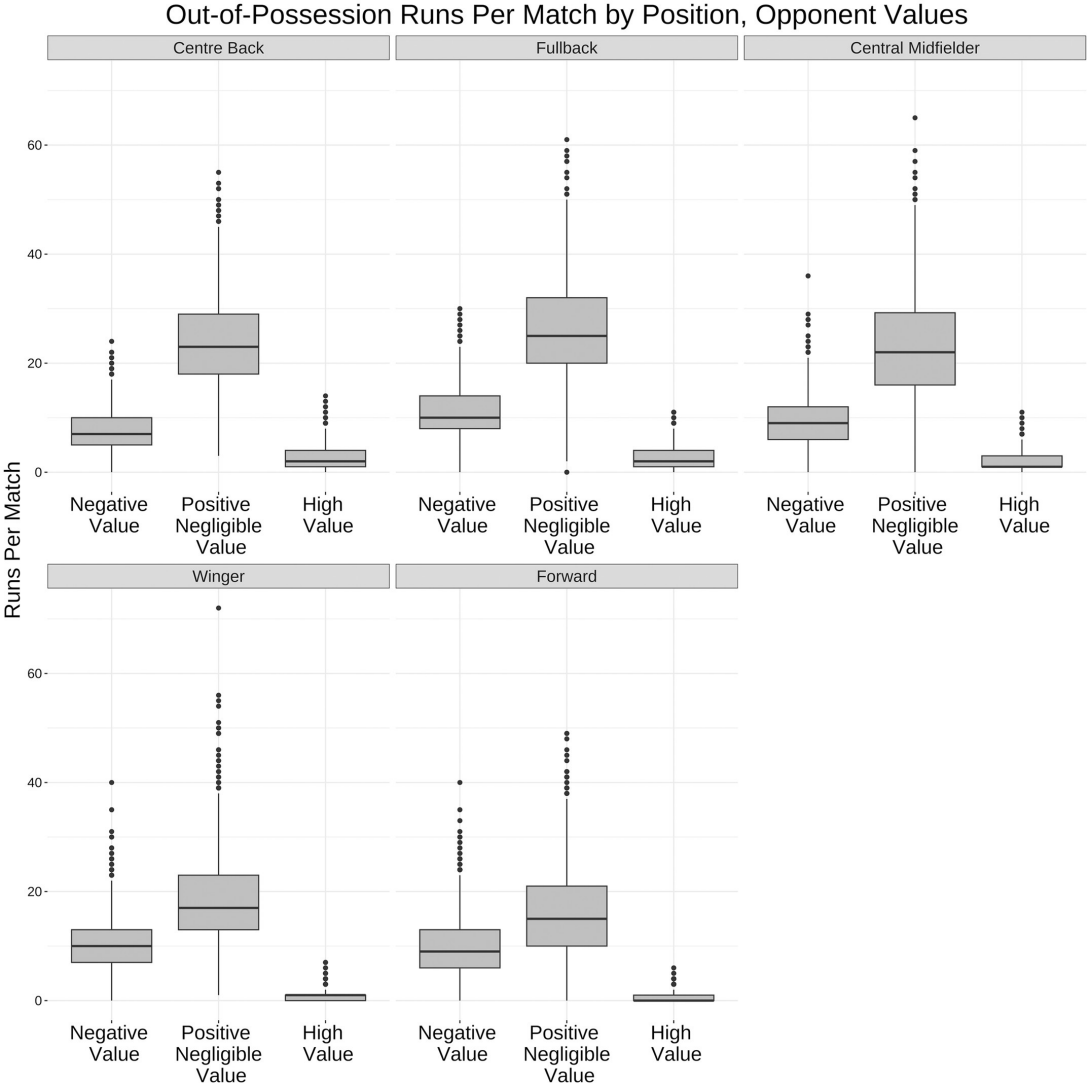
Box and whisker plot for out-of-possession run value buckets by position. Box and whisker plot (horizontal line showing median, box showing 25th and 75th percentiles, vertical line showing highest and lowest points within 1.5 of inter-quartile range) showing the difference in run value by positions out-of-possession. Centre backs and fullbacks make the most runs while their opponents are accruing high value.

Figs 14 and 15 breakdown runs by position and value. For in-possession runs forwards and wingers make the most high value runs whereas centre backs and fullbacks make the most high speed runs out-of-possession while their opponents are accruing value.

### Player-game case study

Categorising high speed runs based on how much value the in-possession team accrues helps to answer more general questions about player movement in football, but can also be used in an applied setting at a much more micro game or game-player level. The following case study provides an example.

**Fig 16:**
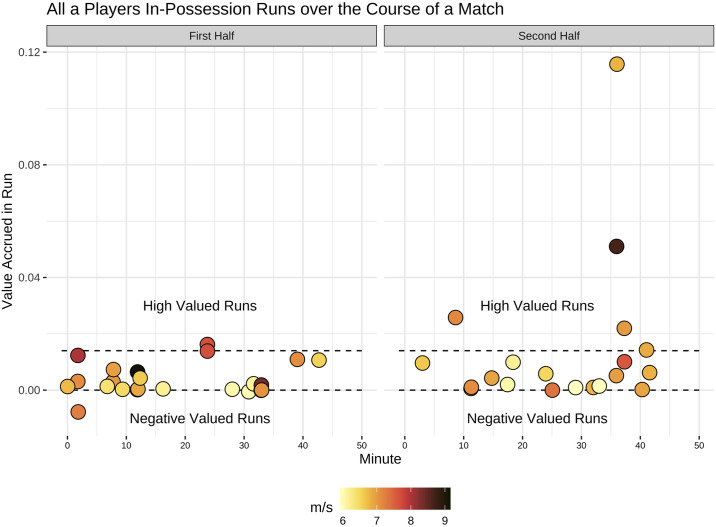
Sample player’s high intensity runs by match minute, value and speed. Timeline of all a player’s in-possession runs over the course of a match. The colour scale shows the speed of the run and the y-axis position shows the value of the run. This shows the majority of the player’s high value runs came later in the game and their negative value runs all came earlier in the match.

Fig 16 shows all the in-possession runs a player made over the course of a match with the colour corresponding to speed, the y-axis value accrued and the x-axis game time.

Most post-match physical data reports will include a summary of the player’s total number of high speed efforts alongside things like distance covered, top speed reached etc. Table 4 shows how dividing runs by in/out-of-possession and value accrual adds additional context.

**Table 4:** The sample player’s run summary. This table demonstrates the additional level of detail in a player’s run profile by using value accrued numbers to analyse the players runs rather than just reporting the total of 80 high-intensity efforts.

	In-Possession			Out-of-Possession		
Values	High	Negligible	Negative	Opponent High	Opponent Negligible	Opponent Negative
Player	6	34	3	3	15	15

## Discussion

This study introduces a new approach to assigning value to player high speed runs in football. Relationships between movement metrics and value are presented on a run-by-run basis, with the effect of player position and team coordinated movements also explored.

### Interpreting results

#### Value and movement profile metrics

The positive associations between value accrued over the course of a run and speed (and to a lesser extent acceleration) are intuitive from a football tactics point of view. There are two potential directions of correlation that could describe this result:

1. Players are typically running fastest and expending energy when they see an opportunity to score, therefore the high probability of the in-possession team scoring encourages players to try and take advantage and increase their speed. These moments are often moments of disorganisation for the defensive team so it also explains why this trend would be mirrored for the out-of-possession team as they try to recover.
2. It may also be the case that higher speed or acceleration runs by the in-possession team are actually causing higher levels of disorganisation in the out-of-possession team and leading to high goal scoring probabilities.

These results also align with similar studies at a more macro scale. Hoppe et al 2015 [40] found positive correlations between in-possession team running totals and final points accumulated in a season as well as negative correlations between out-of-possession team running totals and final points. Schulze et al 2021 [41] identified counter attacks (where defensive disorganisation is high) are the types of attacks that have the highest goal scoring rate, and that defensive team high speed running in the minute before a goal is scored is associated with higher goal scoring rates.

The negative correlation between value accrual and tortuosity is also intuitive from a tactical lens. Similar to speed and acceleration the causation could run in either direction but when there is a higher chance of scoring more players will run directly towards the goal that is being threatened which will result in more straight line runs with lower tortuosity scores.

The relative magnitudes of these affects in and out-of-possession also have some tactical implications suggesting the speed and acceleration of the runs by the attacking team are more closely associated with higher value generation by the attacking team, while less tortuosity or straighter runs by the defensive team is more closely associated with higher value generation by the attacking team (these straight line runs may be interpreted as recovery runs back towards the team’s own goal).

#### Value and coordination

The positive association between multiple players on the same team making high intensity runs and value accrual of those runs suggests one of two explanations.

1. The coordination of player movement of the in-possession team results in a higher probability of scoring—ie. Multiple players coordinating their timing of movement results in more effective attacking moments.
2. In response to a goalscoring moment occurring multiple players make attempts to either provide support on the in-possession team or recover on the out-of-possession team.

The results above suggest a positive relationship between within-team movement coordination and value which adds new insight to some of the higher level trends identified around player coordination and final outcomes. Research in player coordination in both football and other sports have analysed team coordination [42–44] as well as subgroup and dyad coordination [45]. Folgado et al 2018a [46] explored team movement and found that out-of-possession teams exhibit lower movement synchronisation when losing, however unlike the findings in this research this result cannot be directly tied to the moments which led to the team losing. Folgado et al 2018b [47] found that tactical synchronisation between player dyads increased throughout the course of a team’s pre-season which suggests that increasing synchronisation was a focus for the team; a focus would be supported by the positive association between goal scoring probability and coordination identified in this research. Gonçalves et al 2018 [48] identified drop-off in player coordination over the course of a match, particularly for forwards suggesting some mental fatigue, alongside the finding that in-possession coordination is associated with high value; this may have implications for late-game tactical decisions including substitution patterns.

This research only analysed coordination from a speed and timing dimension, but a possible extension could be to examine path similarity [49]. Marcelino et al 2020 [49] created a metric looking at path similarity between concurrent movements and found that players who consistently took different paths than their teammates were associated with higher market/transfer values. This suggests that while coordinating movement timing may be associated with higher value there may be benefits to varying the movement trajectory itself.

#### Value and position

This analysis of the interactions between position, value accrual and high intensity runs brings a new dimension to the existing research on the significant relationships between running statistics and position [26–28]. The positional distribution of value accrued in high intensity runs by positions roughly aligns with the roles of each position.

The defenders (centre backs and fullbacks) make more high intensity runs while their opponents are accruing value and much fewer while their own team has a high probability of scoring. The distribution of centre midfielders is more evenly split between high intensity runs while both teams are accruing value. Whereas the attacking positions (wingers and forwards) have more high intensity runs while their team is accruing value. The even distribution of centre midfielder runs while both teams are accruing value (in and out-of-possession) aligns with the finding in Carling et al 2012 [50] that this position group most often makes high intensity runs separate by short recovery times (≤ 20 seconds).

From a tactical perspective it is also notable that there is a more even distribution of high speed runs during moments of high value by the out-of-possession team than the in-possession team. Suggesting that while defending the entire team is asked to make more of an effort whereas in attacking moments the burden falls mostly on the attacking players. This last finding may offer an explanation for the results in research such as Bloomfield et al 2007) [26] and Di Salvo et al 2010 [27] which show midfielders and forwards spend more time sprinting than defenders; namely that they are required to make high speed runs both when their team and the opponents are in high value moments.

#### Case study insights

Referring back to the case study results there are several insights that can be gleaned by combining the high speed run numbers with value accrued that serve as an example for how this type of reporting could be useful in a practical setting.

Looking at the line graph in Fig 16 there are two trends that stand out: the player generated considerable high value and high speed runs in the period between minute 35–40 of the second half, and the player made one high speed run early in the match that resulted in a negative value accrual for his team. These are two key moments where using this lens of analysis would allow a coach or fitness coach to narrow down on potentially important moments in the match to review with the player or team.

Instead of merely reporting the player made 80 high intensity runs there are a few extra insights gleaned by looking at the advanced division of runs in Table 4. One of the key take-aways from this summary is that while making high intensity out-of-possession runs the opposition accrued a negative value 15 times in 33 runs. This may suggest that the player had an impressive defensive performance and was able to impede the opposition’s attacks with his movement out-of-possession.

### Limitations and extensions

The main limitation of this paper is an attribution problem, which is a problem that plagues many value models [51] In event level models only one player is making an action at a time, so the entire value goes to that player [12, 14] or it is modified to attribute value to different players in the chain [51]. As outlined earlier with the coordination of runs multiple players can be making runs at the same time and they all receive the total team value generated. This means that if the team is accruing a high value and a player is making a run that has minimal effect on this value, they will still accrue the entire team value. This is not an issue for analysing broad trends as this research does but makes it impossible to assign causality to these runs. Which leads to multiple interpretations of causal directionality which is evident by the analysis in of the movement profile metrics.

One potential approach to assign value to individual runs is by looking at the space generated by runs or player gravity in dragging defenders out-of-possession as demonstrated in Fernandez and Bornn 2018 [21]. However, space generation is only one possible way a run may generate value, only looking at the space generated by a run may ignore other ways in which a high speed run has generated value for the in-possession team.

The other main extension would be to extend the analysis to more movement types, not just high intensity runs at a predetermined cut-off. The uniform 5.5 m·s-1 cut off used to determine high speed runs in this paper could be adapted based on gender [52, 53] or even individualised to each player [54, 55]. However the problem of extending this analysis to all movement types is tied to the previous problem of attribution because if all player movement was assigned the value accrued by the team then every player would accrue the same value as the team while they were on the pitch no matter what type of movement they were making.

### Applications

There is little consensus in professional sport on the association between running metrics and results on an aggregate level [56–59]. This in part is leading to the calls for contextualization of these runs [22, 23], however even with tactical contextualization there is still the question of actual value to team performance. By directly tying runs to a value model teams can identify which players and runs are leading to positive outcomes for the team from a goal probability standpoint.

The approach outlined in this paper gives coaches and performance staff the tools to identify how effectively players are expending their energy by putting values on their high speed runs. This could be used as a coaching tool to guide players towards more effective running patterns throughout the games or player/team evaluation tools to identify how effective players are at expending their energy efficiently. In the long term, these tools could be used to design more effective training sessions and program design.

## Conclusion

This paper uses a goal-probability value model to assign values to high speed runs both in and out-of-possession. The value of these runs is then analysed across three metrics from the sport science space (speed, acceleration and tortuosity), as well as the effects of run coordination and player position. The framework applied shows how one of the more popular tools from sports analytics (expected value) can be used to add additional insight to sport science metrics and add value for practitioners.
